# Abrupt switch to migratory night flight in a wild migratory songbird

**DOI:** 10.1038/srep34207

**Published:** 2016-09-26

**Authors:** Daniel Zúñiga, Jade Falconer, Adam M. Fudickar, Willi Jensen, Andreas Schmidt, Martin Wikelski, Jesko Partecke

**Affiliations:** 1 Max Planck Institute for Ornithology, Am Obstberg 1, D-78315 Radolfzell, Germany; 2 University of Konstanz, Department of Biology, D-78457 Konstanz, Germany; 3 University of Glasgow, Institute of Biodiversity, Animal Health and Comparative Medicine College of Medical, Veterinary & Life Sciences, G12 8QQ, Glasgow, UK; 4 Department of Biology, Indiana University, 1001 East Third Street, Bloomington, IN, 47405, USA; 5 Max Planck Institute for Ornithology, D-82319 Seewiesen, Germany

## Abstract

Every year, billions of wild diurnal songbirds migrate at night. To do so, they shift their daily rhythm from diurnality to nocturnality. In captivity this is observed as a gradual transition of daytime activity developing into nocturnal activity, but how wild birds prepare their daily rhythms for migration remains largely unknown. Using an automated radio-telemetry system, we compared activity patterns of free-living migrant and resident European blackbirds (*Turdus merula*) in a partially migratory population during the pre-migratory season. We found that activity patterns between migrant and resident birds did not differ during day and night. Migrants did not change their daily rhythm in a progressive manner as has been observed in captivity, but instead abruptly became active during the night of departure. The rapid shift in rhythmicity might be more common across migratory songbird species, but may not have been observed before in wild animals due to a lack of technology.

Every year billions of songbirds migrate at night, largely hidden from human eyes. Migrant birds perform migratory nocturnal flights, presumably to overcome energetic constraints by flying in cooler and more laminar air^1^, to reduce their predation risk during migration^2^ and to use celestial cues for orientation^3^. In order to migrate over long distances, migratory birds undergo complex changes in several physiological and behavioural traits before and during migration which are often referred to as “migratory syndrome”^4^. To reach the state of readiness for prolonged migratory flights requires dietary changes, involving hyperphagia to increase accumulation of fat as energy storage^5^, changes in enzyme activities associated with energy metabolism^6^, and hypertrophy of flight muscles^7^. In terms of behaviour, many diurnal migratory songbirds need to adapt their daily rhythms by adding a nocturnal phase of activity when they perform their migratory flights at night.

Under laboratory conditions, migratory behaviour of nocturnal migrant bird species is expressed by nocturnal locomotor activity (*Zugunruhe*) during the autumn and spring when migration occurs in the wild^8^,^9^,^10^. *Zugunruhe* is composed of a set of stereotyped behaviours mostly characterized by wing whirring but also includes hopping and fluttering^11^. The amount of *Zugunruhe* has been used as a proxy for the propensity of individuals to migrate^12^ and has been related to genetic, physiological and behavioural aspects of bird migration^8^,^9^,^10^,^13^,^14^,^15^. The seasonal change in rhythmicity from diurnality to nocturnality that leads to the full expression of *Zugunruhe* is assumed to be controlled by endogenous circadian rhythms externally entrained by changes in photoperiod (reviewed in refs 16 and 17). It has been assumed that two circadian oscillators–one controlling the daytime activity and the other controlling the night-time activity–are the main components of the avian circadian clock that triggers migratory nocturnality^18^,^19^. During migration seasons, the oscillators seem to slowly uncouple and stabilize in antiphase^18^,^19^ (reviewed in ref. 16). This process can be observed as an evening or early morning component of activity which separates from the daytime activity and slowly moves into the night^18^. This shift in activity can be visualized as a gradual increase of night activity over time^18^ and has been observed in several bird species e.g. European starlings (*Sturnus* vulgaris)^20^, bramblings (*Fringilla montifringilla*)^21^, garden warblers (*Sylvia borin*)^9^, common quail (*Coturnix coturnix*)^22^ and European blackbirds. In captive European blackbirds, a gradual onset of nocturnality occurs when an early morning component of activity slowly moves into the night (Fig. 1b). Individuals have previously been classified as migrants or residents based on the amount of nocturnal restlessness displayed^23^,^24^ (Fig. 1a,b).

Although the onset of *Zugunruhe* and its underlying mechanisms have been well-studied under laboratory conditions, little attention has been devoted to how migratory birds change their daily rhythm from day- to night-time activity before their migratory journeys in the wild. A major limitation to study such migration-associated behaviours in free–living songbirds has been the lack of suitable tracking technology. Recent technical advancements have produced small and long-lived tracking devices which have led to increased research efforts investigating activity patterns of animals in the wild^25^,^26^,^27^,^28^,^29^,^30^. Reports exist that during the pre-migratory phase, juvenile Eurasian reed warbler (*Acrocephalus scirpaceus*) perform local flights around the breeding grounds^31^. Furthermore, local nocturnal flights have also been described in Swainson’s and Hermit Thrushes (*Catharus ustulatus* and *Catharus guttatus*, respectively) but in this case, during migration at stopover sites^32^. However, studies so far have not focused on measuring activity at a high temporal resolution to elucidate how individuals of a diurnal bird species adjusts their daily rhythm in preparation for nocturnal migration.

To test how free-living migratory songbirds extend their diurnal life style to include an additional nocturnal component while performing their migration, we studied a partially migratory population of European blackbirds using an automated radio telemetry system^28^,^33^,^34^. Partial migration, where both migrants and year-round residents are present within the same population^35^, provided us with an ideal study system to directly compare differences in behaviour between migrant and resident individuals^36^. In the population we investigated, migrants show specific characteristics that are known for classical obligate migratory bird species. Migrants accumulate fat during the pre-migratory phase as preparation for migration, while residents do not fatten up during this period of time^37^. Furthermore, migrant European blackbirds migrate at night. Ground radio tracking data collected while following birds during their first night of the autumn migration using tracking cars show that departures from the breeding grounds occur during the first half of the night. During that first night of migration, birds fly non-stop >200 km until they reach their first stopover site, and land just before sunrise (unpublished personal data). Data obtained from light-based geologgers indicate that migrants in this population overwinter on average 800 km west-southwest from the breeding grounds^38^.

To test whether European blackbirds prepare for migration by increasing their locomotor activity, we compared day and night activity patterns of radio-tagged migrant and resident individuals of a partially migratory population of European blackbirds during the pre–migratory phase.

## Results

All migratory birds departed between 23 September and 26 October (mean departure date: 14 October). All departures occurred before midnight, with mean departure time of 2.2 hours after civil dusk (min = 0.1 hour, max = 4.7 hours). Migrants were active 36.76 ± 17.48% (mean ± SD) during daytime, and residents were active 33.56 ± 17.36% (mean ± SD). During nights, migrants were active 2.28 ± 7.28% (mean ± SD) and resident birds 1.86 ± 6.86% (mean ± SD). Figure 2 shows the mean activity value of each half hour interval of resident and migrant individuals during the seven days and nights before the departure of migrants. During the seven days prior to departure, total day and night activity of migrant and resident European blackbirds did not differ (GAMM: estimate ± SE = −0.2088 ± 0.12, z-value = −1.70, *P* = 0.089; Fig. 3). Mean predicted activity profiles of resident and migrant individuals exhibited similar curves and amplitudes over time (Fig. 3). Only during the night of departure did migrants show higher activity levels compared to residents during the first half of the night (Figs 2 and 3). This is likely caused by the actual take-off of the migrants and confirms that our methods were sufficient to detect nocturnal activity differences among individuals.

To test if migrants increased their activity levels during the days before departure, we combined individual activity data during the seven pre-departure days into one 24 h period. We ran a generalized additive mixed model (GAMM; referred in the method section as “hourly GAMM”) to detect possible hourly differences in activity and to account for the diel variation in activity. The hourly GAMM did not find that activity levels were different between migrant and resident individuals (GAMM: estimate ± SE = −0.147 ± 1.122, z-value = −1.20, *P* = 0.23). We then extracted the residuals of this GAMM and tested separately the residual values of daytime and night-time observations. The residual analysis of daytime observations found that the interaction between time (days before departure) and the migratory status was not significant (LMM: estimate ± SE = 0.006 ± 0.010, t-value = 0.63, *P* = 0.526), which implies that daytime activity did not differ between the groups over time. Similar results were obtained for the night-time observations: night-time activity did not change over time (LMM: estimate ± SE = −0.008 ± 0.022, t-value = −0.382, *P* = 0.702).

Finally, a comparison between the levels of nocturnal locomotor activity displayed by both groups in mid-August and during the pre-migratory phase was performed. The amount of nocturnal activity displayed by migrants and residents, was not different when we compared mid-August to the period prior to migration (LMM: estimate ± SE = −0.369 ± 0.525, t-value = −0.703, *P* = 0.487).

## Discussion

**Figure 1:**
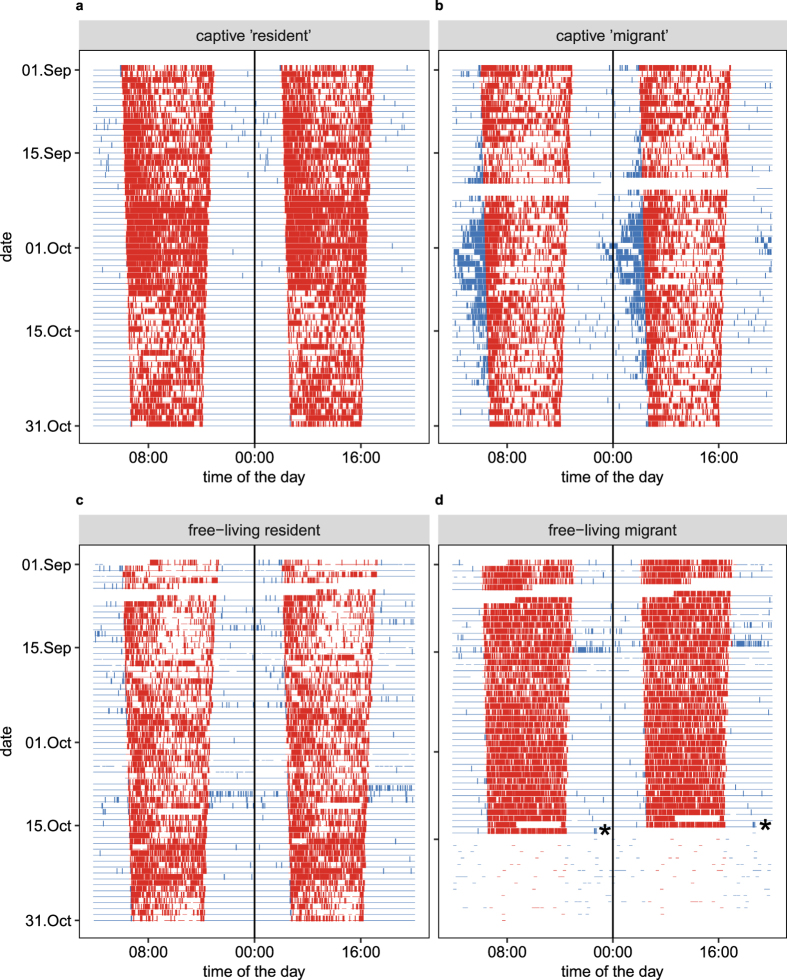
Double-plotted actograms (48 hours) showing activity of a ‘‘resident’’ captive, “migrant” captive, free-living resident and free-living migrant European blackbirds (*Turdus merula*) during autumn. Captive “resident” (**a**) and captive “migrant” (**b**) were exposed to natural photoperiod during the autumn (Sep. 1–Oct. 31). These two actograms were plotted using raw data from Partecke & Gwinner (2007). In the case of the captive “migrant” (**b**), around mid-September a morning component of activity moves gradually into the night-time, developing nocturnal activity (*Zugunruhe*). *Zugunruhe* peaks the night of October 2. Activity of a free-living resident bird (**c**) was recorded using the automated telemetry system (ARU) continuously from the autumn until the consecutive spring. (**d**) Activity of a free-living migrant bird was recorded also using the ARU, during the autumn until its departure. The departure time of the free-living migrant is indicated by *. After departure, the ARU showed some false positive inactivity due to noise in the recording. Day and night time activity coloured red and blue, respectively.

**Figure 2:**
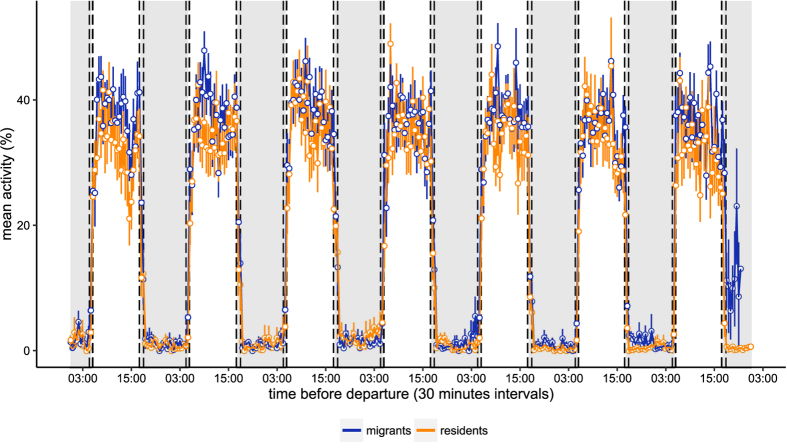
Activity pattern of migrant and resident birds seven days before the departure of migrants. Mean activity % and standard error of migrant (blue) and resident (orange) European blackbirds (*Turdus merula*) in 30 minute intervals four days prior to departure. White and grey backgrounds represent mean day and night-time respectively. Dashed vertical lines represent the variation in dawn and dusk onset during the time of the study. Sample size: 21 migrant individuals (11 females, 8 males and 2 unknown sex birds; 2 individuals with multiple departure events) and 23 resident individuals (3 females and 20 males).

**Figure 3:**
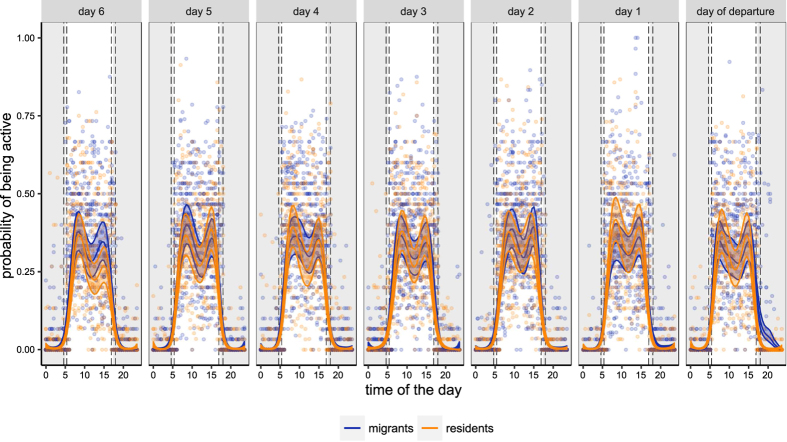
Results of the Generalized Additive Mixed Model (GAMM) on daily activity seven days before the departure of migrants. Predicted daily activity pattern by GAMM of migrant (blue) and resident (orange) individuals. Solid lines represent the mean fitted values of the model (smooth term) with 95% confidence interval (shaded area). Dots correspond to the raw observations. White and grey rectangles represent mean day and night time respectively. Dashed vertical lines represent the variation of dawn and dusk onset during the time of the study.

Our results provide strong evidence that at the onset of autumn migration, European blackbirds rapidly shift from an exclusively diurnal activity pattern to migrate at night without any previous changes in their daily rhythm. We used an automated radio-telemetry system, which accurately depicted day and night cycles of activity in our wild European blackbirds. Even though, the activity patterns were biological meaningful, we also observed bouts of activity during the night in both resident and migrant individuals without a consistent pattern (Fig. 1). These bouts of “nocturnal activity” could be false positive measurements. Artefacts due to atmospheric conditions (stormy weather) or electric noise that can appear as “activity” were observed in these readings. However, there were cases where we observed short bouts of activity during the night that resembles the diurnal pattern of activity. In this case birds could have changed their roosting location or simply moved, for instance, due to presence of predators or environmental disturbances.

Very limited knowledge about the nocturnal life of birds is available and only a handful of studies have focused on this topic. Nocturnal local movements and foray behaviour have been described to take place during the breeding season in the yellow-breasted chat (*Icterina virens*)^39^ and in juvenile Eurasian reed warblers. The latter exhibit nocturnal flight during the pre–migratory phase to either develop navigational skills for migration or as juvenile dispersal^31^,^40^. Nocturnal flights to perform local movements have been recorded in Swainson’s and Hermit Thrushes during migration at stopover sites^32^. In contrast to these studies, our results clearly indicate that neither locomotor activity nor exploratory flights during night were exhibited by migratory European blackbirds prior to their migratory departure. These results support the idea that no significant amount of nocturnal locomotor activity or nocturnal exploratory flights are developed as preparation for migration in the wild. We only detected an increase of activity in the migratory birds during the night of departure, which largely resulted from detection of actual take-off behaviour on our automated receiving system. These data provide a positive control that our recording system would have detected increased nocturnal activity if present. We also found similar low levels of nocturnal locomotor activity during August. This is well outside the migratory period and when European blackbirds are in their main moulting phase compared to the pre-migratory period. This result indicates that the amount of nocturnal locomotor activity displayed by birds of both groups is not related to the migratory season.

The rapid shift to nocturnal activity in the night of departure with all departure events occurring before midnight, appear not to match what has been observed in previous laboratory studies of nocturnal activity in migratory songbirds. In the case of European blackbirds, there is a gradual progression of an early morning component of activity into the night, at the onset of *Zugunruhe* whenever birds are kept under natural photoperiod (Fig. 1b). The onset of nocturnality in birds during the migratory season is controlled by the circadian clock. When the two oscillators that control day and night activity respectively, slowly uncouple^18^,^19^, the gradual shift in activity is observed in the lab. In the wild, the rapid shift from daytime activity to nocturnal activity just during the night of departure may indicate that under natural conditions, the circadian clock might be affected by extrinsic factors. For example, a masking effect of atmospheric conditions could influence the decision to initiate the migration and suppress the expression of nocturnality prior to departure. Birds could simply wait for the right conditions to migrate and then decide to start the migratory journey very shortly afterwards. There is evidence that nocturnal migrants rely on atmospheric conditions to initiate their migratory journeys^41^,^42^,^43^. Furthermore, predator avoidance as an ultimate mechanism may also influence the expression of nocturnal activity prior to migration. Being active at night could alert potential predators and increase the risk of predation. An alternative explanation for the possible discrepancy between lab and field data could be that a gradual increase of nocturnal activity does not happen during pre-migration but rather after departure during the course of migration. This process could be reflected by a shift in the departure time resulting in earlier departures during subsequent nights. The gradual shift could also be reflected by an increase in flight duration or efficiency resulting in a systematic increase in distance travelled each night during migration. This idea seems less plausible because during radio tracking of the first night of migration (unpublished data), migrants fly non-stop throughout the night. However more data are needed to fully test this hypothesis. Future studies using devices that quantify and store locomotor activity may be able to test whether migrants increase their flight duration or efficiency during their migratory course.

Our results provide no evidence for different activity pattern between migrant and resident European blackbirds during the pre–migratory phase in the wild, both during day and night. The lack of a difference in daytime activity between migrants and residents birds is interesting because one would expect that migrant individuals would show higher activity compared to resident birds, as a consequence of an increased foraging effort (hyperphagia)^44^. One possible explanation for the lack of a difference could be that resident birds also have to accumulate energy reserves in view of the upcoming winter. However, previous studies have shown that migrants in this population accumulate fat during the pre-migratory phase while residents do not^37^. Moreover, residents birds accumulate fat later in the year when the winter already started^45^. Another alternative is that the two groups have a similar total activity budget during the day but invest their time fulfilling different requirements. Migrants may invest more of their total time spent active in foraging at the expense of other activities (e.g., anti-predator vigilance). A similar trade-off has been described in ruddy turnstones (*Arenaria interpres*) during the pre-migratory period. Migratory individuals decreased their anti-predator vigilance, whereas non-migratory birds showed no decrease in this activity^46^. We were not able to test this hypothesis mainly due to two limitations of the automated radio tracking system we used: first is not possible to identify specific behaviours and second, the sampling resolution of 1 minute might not be sufficient to estimate the overall activity budget precisely. Furthermore, different foraging strategies between migrant and resident European blackbirds could also explain our results. Both phenotypes would invest similar amount of time foraging but migrant birds would do it more efficiently than the resident counterpart. Foraging efficiency had an effect on the amount and speed of fat deposition during the autumn pre–migratory period in gray catbirds (*Dumetella carolinensis*)^47^. One could assume that foraging is the main activity in the total budget during the pre-migratory phase, at least for migrants, because migrants undergo a pre-migratory hyperphagic phase^5^. Under this assumption the increased fat deposition in the migrants in contrast to the residents, could be explained, at least partly, by a higher efficiency of food utilization by migrants. In garden warblers, food utilization is more efficient during the pre-migratory period in autumn and spring compared with the rest of the annual cycle^48^. Furthermore, optimal diet selection has been also linked with effective fat accumulation before migration with migrants selecting a diet rich in lipids and carbohydrates^49^. It is also conceivable that migratory individuals lower their energy expenditure during the pauses in locomotion, similar to zebra finches in captivity that are exposed to higher workloads^50^.

In this study we focused on the pre-migratory phase. Further development and miniaturization of tracking devices with GPS and accelerometer sensors will make it possible to study the nocturnality of migratory songbirds during their migratory journeys and stop over sites in detail. These future studies could make use of the new technological advances to uncover further mysteries related with the fascinating phenomena of bird migration in the wild.

## Methods

### Ethical Note

All the experimental procedures were performed in accordance with the German regulation on animal experimentation. The experimental protocol was approved by the Ethical Committee of Baden-Württemberg (Regierungspräsidium Freiburg).

### Study population, capture and tagging

The study population of European blackbirds inhabited a mixed coniferous/deciduous forest in south-western Germany (N47°47′, E9°2′). Birds were captured during March and September throughout the years 2009–2012 and 2014–2015 with mist nets. Each bird was weighed (to the nearest g) and subsequently fitted with a radio transmitter (≤2.6 g; Sparrow Systems Fisher, IL, USA) by means of a leg loop harness. Transmitters possessed a battery life of at least one year. Recaptures of previously tagged birds were made during the last six years of the study, allowing old transmitters to be removed and renewed. The combined weight of the equipment was <5% of the mass of the individual carrying it. Leg loop harnesses were constructed of black elastic cord (1 mm thick) in a range of sizes depending on the body weight of each bird (e.g. 120 mm cord for a 75 g bird–140 mm cord for a 90 g bird) to ensure optimal fit. Age and sex of each bird were recorded based on plumage variations^51^. All birds were observed post-release to confirm normal behaviour. For the present study we used 28 males (26 adults, 2 juvenile), 14 females (9 adults, 5 juveniles) and 2 juveniles of unknown sex.

### Automated Receiving Unit (ARU) data collection

For the duration of the study, three to five Automated Receiving Units (ARUs; Sparrow Systems Fisher, IL, USA) attached to mounted H antennas (ATS, Isanti, MN, USA) were stationed at well-exposed sites in the study area to enable continuous reception of radio signals of tagged individuals. ARUs searched for the frequencies of deployed radio transmitters once every 60 seconds, recording values for signal and noise. This enabled the activity of tagged individuals to be monitored on a continuous basis^25^,^28^,^33^,^39^,^52^,^53^. ARUs additionally monitored the activity of static radio transmitters which were attached to stationary posts at the study site to check the normal functioning of the ARUs and to correct for the occurrence of noise.

### Determination of migratory status, departure date and activity

The migratory status of each bird was determined by tracking individuals using manual radio telemetry and continuous ARU monitoring. Birds were manually tracked twice per week using a handheld three element Yagi antenna (AF Antronics, Inc., Urbana, IL, USA) and an AR 8200 MKIII handheld receiver (AOR U.S.A., Inc., Torrance, CA, USA) or a handheld H-antenna (Andreas Wagener Telemetry Systems, Köln, Germany) connected to a Yaesu handheld receiver (Vertex Standard USA, Cypress, CA, USA). Individuals whose signal was not detected by radio tracking were searched for aerially using a Cessna airplane fitted with two H-antennas and two Biotrack receivers (Lotek Wireless Inc., Newmarket, ON, Canada.). These searches covered a minimum of 20 km in radius. Due to the higher detection probability of the radio signal from the sky our aerial search method allowed us to confidently determine the presence or absence of a bird carrying a radio transmitter^37^. If no signal was obtained for an individual after at least two aerial searches, it was defined as a migrant. An individual was defined as resident if it remained in the study site until the beginning of the next breeding season (March). We classified 9 females, 8 males and 4 birds with unknown sex as migrants; and 3 females, 19 males and 1 bird with unknown sex as residents.

To quantify the activity pattern of individual European blackbirds, we used the change (Δ) in signal strength between successive one minute recordings and applied a threshold value of 4.0 dB. An individual was either categorized as active or inactive, depending on whether the change in signal strength was above or below this threshold, respectively. To calculate this threshold, we carried out a calibration experiment in which we randomly distributed 13 radio tags throughout the study site. Each tag represented a tag on an inactive bird. ARUs recorded data from these tags for a total of one week. We then pooled the values of consecutive signal Δ between one minute intervals and used the 99% upper quantile to estimate the maximum variation in signal change occurring in an “inactive bird”. This value was taken as the threshold. Our calibrated threshold value was consistent with those of previous efforts^28^ and this method has been previously used in forested landscape^25^. An individual was either categorized as active or inactive, depending on whether the change in signal strength was above or below this threshold, respectively.

ARU’s have a reception range of 850 meters in our study site, although this value is subject to variation depending on topography, atmospheric conditions and position of the bird relative to the ARU antenna. Whenever the signal was absent but reappeared later, such as when the bird was out of range of the ARU, we classified these periods as ‘unknown’. For this assignment we applied a threshold to the minimum signal strength of −127.0 dB, a value that is 4 dB greater than the mean of the upper 95% quantile of the white noise recorded at the same time. We also assigned periods to be ‘unknown’ when there was less than 10 dB between signal and noise or where the value of the noise was more than −130 dB, indicating high electronic noise in the entire area, such as during thunderstorms.

The raw data were inspected visually to detect and filter artefacts produced by malfunctioning of the ARU. These artefacts were produced presumably by electromagnetic interference, for example, due to stormy weather conditions. We also decided to filter our data based on its quality. Only daily activity budget measurements in which the uncertainty value was under 10% were used for further analysis. We decided to use this conservative approach to be sure that the activity % calculated was a representative measure of the daily activity.

We used R version 3.2.1^54^ to generate and analyse time series of binary activity data. Activity budgets were calculated as the proportion of the total number of minutes a bird was active during a 30-min period. The start of the time series was seven days before a departure event of a migrant occurred and the end of the time series was set to midnight of the night of departure. Before choosing seven days we explored the data by plotting up to 30 days before departure and we did not observe a different pattern of activity between the two groups during this period of time. In this way we aligned 24 departure events from 21 individual migrants (1 individual was included with 2 departures in different consecutive years and another bird was included with 3 departures in 3 consecutive years). The departure time was estimated by an algorithm written in R Version 3.2.1. The algorithm searched for the first time stamp after the signal in the ARU was absent permanently (the point where only unknown values were present). Visual inspection of the data was later used to confirm these estimations.

To compare migrant and resident birds, we randomly paired one migrant with one resident individual that was present at the breeding ground at the same time the departure event occurred. One resident individual was included with two departures in different consecutive years. We generated equivalent time series using data for those resident individuals.

To define day and night-time, we calculated for each day, when the morning and evening civil twilight occurred using the function “crepuscule” from the R package “maptools”^55^. This function estimates for a given date and coordinate when the geometric centre of the sun is 6° below the horizon in the morning (civil dawn) and in the evening (civil dusk). Daytime was defined as period of time between dawn and dusk, and night-time corresponded to the period of time between dusk and the consecutive dawn.

To generate the actograms presented in Fig. 1a,b, we obtained raw activity data of one “captive resident” and one “captive migrant” from the study carried out by Partecke & Gwinner^24^. We did not process these data in any form but use it for visualization purposes and as an example of how *Zugunruhe* is expressed under laboratory conditions.

### Statistical analysis

Statistical analyses were conducted using R 3.2.1^54^. To compare activity levels between migrants and resident individuals, we modelled the daily rhythm of activity of each phenotype during 7 days before the departure of migrants. Given the non-linear nature of the data we fitted a Generalized Additive Mixed Model (GAMM) using the function “gamm4” from the package “gamm4”^56^. The flexibility of these models allows us to fit a non - linear smoothed function (smooth term) which resembles a sinusoid-like curve that describes the day and night rhythmicity of the time series. We modelled the probability of a bird being active using a binomial error distribution and a logit-link function in relation to time of the day and the migratory strategy. The dependent variable activity was expressed as minutes of activity within 30 minutes. The independent variable time of the day was expressed in hours of the day and was included as the main parameter to construct the smooth term of the GAMM. We used the default thin plane regression splines (BS = “tp”) parameter to construct the smooth term. We included the interaction between migratory strategy, day before departure, and time of the day in the smooth term using the argument “by”. This interaction resulted in one independent smooth function being fitted for each group (migrants and residents) and for each day before departure. The choice of seven days helped with model convergence given the high number of parameters needed to be calculated. We also included migratory status as a fixed effect. As random effects we included individual identity for two reasons, first to account for repeated measurements and secondly to add a correlation structure between observations of the same bird that were close in time and therefore account for potential temporal autocorrelation of the time series. Additionally we included an observation level random factor to account for overdispersion of the binomial model. Observation level random factors, where each data point receives a unique level of a random effect that models the extra-binomial variation present in the data, are commonly employed to cope with overdispersion in binomial data^57^.

Given our biased sex ratio of migrant and resident birds, we did not include sex as a fixed effect. We also did not include age as a fixed effect given that we did not have enough juveniles to test for age differences.

For further analyses we ran four post hoc tests in a step wise manner. First, we tested whether the activity changed as time to departure approached. To do so, we performed the following procedure; first, we fitted another GAMM to capture the diel variation of activity. To fit this hourly GAMM we lumped the seven days before migration into a single day of 24 hours. The number of minutes active over a 30 minutes interval was modelled in relation to the time of the day in minutes as a smooth term using a binomial error with a logit-link function. The smooth term was constructed using the cyclic cubic spline “bs = cc”, to account for the cyclic nature of the data. Migratory status was included as a fixed effect and we also included the interaction between time and migratory status in the smooth term using the argument “by”. Random effects in this model were individual identity and year to account for repeated measurements and to add a correlation structure between of observations of the same bird that were close in time. We then extracted the residual values from the hourly GAMM model and split them into day and night time observations. We calculated the mean value for daytime and night-time per individual per day before departure. This was to test whether the remaining variation left in the residuals of the hourly GAMM after accounting for the diel variation of activity, could still contain unexplained differences between migrant and resident individuals or could contain a pattern that suggested an activity change (increase or decrease) as departure approached. Using the residual values of the hourly GAMM we fitted two separate linear mixed models (LMM), one for daytime and another for night-time values respectively. In both models, the residual variation was modelled in function of time as the days before departure, migratory status and the interaction between days before departure and migratory status. Random effects in both models included individual identity and year.

Finally, the last analysis aimed to test whether the amount of nocturnal activity displayed by individuals of migrants and residents prior to the onset of migration was comparable to activity levels displayed outside the migratory season. We compared time series of the pre-migratory season with time series generated in summer. To do so we calculated the mean night activity of each individual during the ten days before departure and the mean night activity of each individual during seven days during mid-August (August 13 to 20 of each year). We then compared them using an LMM approach. Because during mid-August European blackbirds are moulting, we would not expect any nocturnal activity related to migration. The dependent variable in this LMM was the log transformed mean activity value (to account for the non-normal distribution of the variable (right-skewed)). Fixed effects included period of time as a factor (August or pre-migration), migratory status and the interaction of both. Random effects included individual identity and year to account for repeated measurements.

Before drawing conclusions in all the models, a visual residual analysis was performed to check for homogeneity of the variance, model assumptions, temporal autocorrelation and model fit. Significance was considered when p-values were smaller than alpha (0.05) or when 95% confidence intervals predicted by the model did not overlap between groups.

## Additional Information

**How to cite this article**: Zúñiga, D. *et al.* Abrupt switch to migratory night flight in a wild migratory songbird. *Sci. Rep.* **6**, 34207; doi: 10.1038/srep34207 (2016).
