# Corrigendum: Abrupt switch to migratory night flight in a wild migratory songbird

**DOI:** 10.1038/srep35327

**Published:** 2016-12-09

**Authors:** Daniel Zúñiga, Jade Falconer, Adam M. Fudickar, Willi Jensen, Andreas Schmidt, Martin Wikelski, Jesko Partecke

Scientific Reports 6: Article number: 3420710.1038/srep34207; published online: 09 26 2016; updated: 12 09 2016

This Article contains an error in the legend of Figure 2.

“Mean activity % and standard error of migrant (blue) and resident (orange) European blackbirds (Turdus merula) in 30 minute intervals four days prior to departure”.

should read:

“Mean activity % and standard error of migrant (blue) and resident (orange) European blackbirds (Turdus merula) in 30 minute intervals seven days prior to departure”.

